# Monocyte to high-density lipoprotein ratio is associated with mortality in patients with coronary artery diseases

**DOI:** 10.1186/s12872-023-03461-y

**Published:** 2023-09-11

**Authors:** Gaiqin Pei, Rui Liu, Lu Wang, Chengqi He, Chenying Fu, Quan Wei

**Affiliations:** 1 Department of Rehabilitation Medicine and Institute of Rehabilitation Medicine, West China Hospital, Sichuan University, Chengdu, Sichuan PR China; 2 Key Laboratory of Rehabilitation Medicine in Sichuan Province, Chengdu, Sichuan China; 3 West China Hospital, National Clinical Research Center for Geriatrics, Sichuan University, Chengdu, Sichuan China; 4 Aging and Geriatric Mechanism Laboratory, West China Hospital, Sichuan University, Chengdu, Sichuan China

**Keywords:** Monocytes, HDL, MHR, Coronary heart diseases, Death, Cohort study

## Abstract

**Background:**

Whether the monocyte to high-density lipoprotein ratio (MHR) is associated with the prognosis of coronary artery disease (CAD) is inconclusive.

**Methods:**

Patients with CAD were enrolled and their data were collected. Blood was sampled within 24 h after admission. Multivariate Cox regression analysis was performed to determine the relationship between the MHR and all-cause mortality as well as complications during hospitalization.

**Results:**

We included 5371 patients in our cohort study. Among them, 114 (2.12%) patients died in hospital. MHR was independently associated with all-cause mortality (hazard ratio [HR], 1.81; 95% confidence interval [CI] 1.35, 2.42), cardiovascular mortality (1.69; 1.17, 2.45) and non-cardiovascular mortality (2.04; 1.27, 3.28). This association was only observed in patients with hypertension (P for interaction = 0.003). Patients with higher MHR levels also have a higher risk of complications, including infection, pneumonia, electrolyte disturbance, gastrointestinal bleeding, multiple organ dysfunction syndrome, and disturbance of consciousness. The receiver operating characteristic (ROC) analysis showed that the MHR had higher prognostic values than monocytes and high-density lipoprotein.

**Conclusion:**

MHR was an independent predictor of all-cause mortality and in-hospital complications in patients with CAD, especially in patients with hypertension.

## Introduction

Coronary artery disease (CAD) is the leading cause of death worldwide [1]. In China, the prevalence and mortality of CAD are continuously increasing [2]. In 2013, the number of cardiovascular disease deaths reached 3.72 million [3]. In 2018, the mortality rate of Chinese urban residents was 120.18/100,000, and that of rural residents was 128.24/100,000 [4]. A study on the global burden of disease suggested that CAD deaths in China accounted for about 38.2% of the global increase in CAD deaths from 1990 to 2017. Compared with other countries, China experienced the largest increase in deaths during this period [5]. Considering that the outcomes of patients with CAD are still unsatisfactory, new markers are needed to identify patients with a high risk of mortality in order to accurately optimize the administration of management.

Recently, inflammation was reported to promote the progression of coronary atherosclerosis. Inflammatory biomarkers in response to the development of atherosclerosis have sparked interest. Inflammation and lipid deposition lead to vascular endothelial damage, and promote the formation and rupture of atherosclerotic plaques, which play important roles in the occurrence and development of CAD [6]. Circulating inflammatory cells, such as leukocytes and their subtypes, were associated with unfavorable outcomes in patients with CAD [7]. Previous studies have shown the different roles of monocytes and high-density lipoprotein (HDL) during this inflammatory process. For example, Nozawa et al. found that a high monocyte count was closely associated with atherosclerotic plaque progression in patients with ST-segment elevation myocardial infarction [8]. However, higher levels of circulating HDL levels were associated with a lower risk of major adverse cardiovascular events, all-cause mortality, and cardiac death in patients with CAD [9].

Therefore, the monocyte to HDL ratio (MHR) calculated from monocytes with pro-inflammatory effects and HDL with anti-inflammatory effects has emerged as a novel indicator of inflammatory responses in vascular diseases [10]. MHR indicates inflammatory and anti-inflammatory effects, and is potentially involved in the inflammatory process, atherosclerosis progression, oxidative stress, and endothelial dysfunction [11]. Exploring the role of the MHR may be beneficial for the risk stratification and prognostic evaluation of patients with CAD. High MHR was one of the independent predictors of cardiovascular events in patients with chronic kidney diseases [12]. Additionally, MHR was associated with disease severity and bare-metal stent restenosis in patients with CAD [13, 14].

Previously, there were 2 studies about the role of MHR in short-term mortality, but they only focused on patients with ST-segment elevation myocardial infarction [15, 16]. Whether there exists a positive association in other types of CAD is unknown. Moreover, an important limitation of these 2 previous studies was that they did not compare the prognostic values of MHR with monocytes and HDL, and thus the insufficient information might weaken their power to determine the value of using a composite index (MHR) than sample single indexes (monocytes and HDL). Based on the abovementioned pieces of evidence, we aimed to investigate the association between MHR and short-term outcomes in patients with CAD.

## Methods

The retrospective cohort study was based on consecutively recruited patients with CAD in West China Hospital. The project was approved by the Scientific Research Department and conformed to Declaration of Helsinki. We included patients diagnosed with CAD who were admitted from January 2016 to December 2020. CAD is diagnosed as stenosis of more than 50% in at least one coronary artery including the left main, left anterior descending, left circumflex, right coronary, or their main branches by coronary angiography [17, 18]. We included patients aged more than 18 years and excluded patients with insufficient data in laboratory tests, and patients diagnosed with severe hematologic disease, infection, tumor, severe liver and renal failure on admission.

### Data collection

We recorded patients’ information by structured forms including: demographic statistical data, vascular risk factors, laboratory tests, in-hospital complications, and in-hospital mortality. Heart failure was diagnosed with an impairment of ventricular filling and ejection dysfunction, with dyspnea, fatigue, edema, and limited physical activity as the main clinical manifestations [19]. Chronic kidney diseases were diagnosed as a glomerular filtration rate < 90% with a structural or functional impairment of the kidneys due to various causes that persists for 3 months or more. Smoking was diagnosed with taking more than 10 cigarettes per day, for 6 months or more [20]. Drinking was diagnosed with taking alcohol more than 60 g per day, or > 420 g per week, for 6 months or more [20]. All patients were treated according to treatment guidelines [21]. The decision of cardiac surgeries such as percutaneous coronary intervention (PCI), coronary artery bypass graft (CABG), valvular surgery, and radiofrequency ablation were made at the discretion of the cardiologists.

Blood samples were collected from a peripheral vein using ethylene diamine tetraacetic acid (EDTA) tubes within 24 h after admission and analyzed using a Sysmex automated hematology analyzer (Sysmex, Kobe, Japan) and a fully automatic biochemical analyzer (Mindary, BS-820, Shenzhen, China). MHR was calculated as the absolute monocyte counts (10^9^/L) divided by HDL (mmol/L) and reported as 10^9^/mmol.

The primary outcome was all-cause mortality, defined as death due to any reason during hospitalization. The secondary outcomes were cardiovascular mortality, non-cardiovascular mortality, as well as in-hospital complications which were categorized based on medical records. Cardiovascular mortality included death attributed to cardiac arrest, cardiac rupture, cardiogenic shock, and death due to life-threatening heart failure, arrhythmia, aortic dissection, and myocardial infarction [22]. Cardiogenic shock was diagnosed as reduced cardiac output, end-organ hypoperfusion, and hypoxia caused by the impairment of myocardial performance [23]. Non-cardiovascular mortality included all other causes of death such as respiratory failure, multiple organ dysfunction syndrome (MOD), infectious shock, and gastrointestinal bleeding [24].

In-hospital complications included infection, pneumonia, electrolyte disturbance, MOD, disturbance of consciousness, and gastrointestinal bleeding during hospitalization. Infection was diagnosed as local tissue or systemic inflammation caused by bacteria, viruses, fungi, parasites and other pathogens [25]. Pneumonia was defined as an acute infection of the lung caused by different pathogens with symptoms including fever, dyspnea, cough and expectoration [26]. Electrolyte disturbance was defined as abnormal levels of electrolytes, such as hypernatremia, hyponatremia, hyperkalemia, and hypokalemia [27]. MOD was diagnosed as reversible physiologic disorders involving 2 or more organ systems [28]. Disturbance of consciousness was diagnosed as somnolence, stupor, or coma during hospitalization [29]. Gastrointestinal bleeding was diagnosed as blood loss originating from the gut manifested with hematemesis or black stool [30].

### Statistical analysis

We categorized patients into 3 groups according to MHR levels. If data were normally distributed, they were reported as the mean and standard deviation (SD); If data were not normally distributed, they were presented as the median and interquartile range (IQR). Categorical data were expressed as counts and percentages. We compared the differences in continuous data by one-way ANOVA and Kruskal-Wallis H test, and compared the differences in categorical variables by the χ^2^ test or Fisher exact tests.

We used a Cox proportional multivariate hazards regression model to calculate hazard ratios (HRs) and 95% confidence intervals (CIs) of the MHR as continuous data and the MHR as categorical data for the risk of mortality. We first performed univariate analysis to identify variables with a P-value < 0.05. These variables were regarded as potential confounding factors, and were adjusted in multivariable analysis to identify the independent association between MHR and mortality. In model 1, we adjusted for age and sex; in model 2, we further adjusted for other confounders. The linear trend (P for trend) was tested by entering the median MHR value in each categorical group as a continuous variable in the models. To determine the discriminative ability of monocytes, HDL, and MHR, we generated a receiver operating characteristic (ROC) curve and calculated the area under the curve (AUC) for monocytes, HDL, and MHR values to predict the risk of mortality.

We used stratified Cox regression to perform subgroup analyses by variables including age (> 65 and ≤ 65), sex (male and female), hypertension, diabetes, atrial fibrillation, or chronic kidney diseases. The difference between subgroups was inspected by interaction analysis using likelihood ratio tests and the significance of the interaction (p-interaction) was tested. We performed statistical analyses by Stata Version 15.0 (College Station, TX, USA). A two side P < 0.05 was considered statistically significant.

## Results

### Study participants and baseline characteristics

**Fig. 1:**
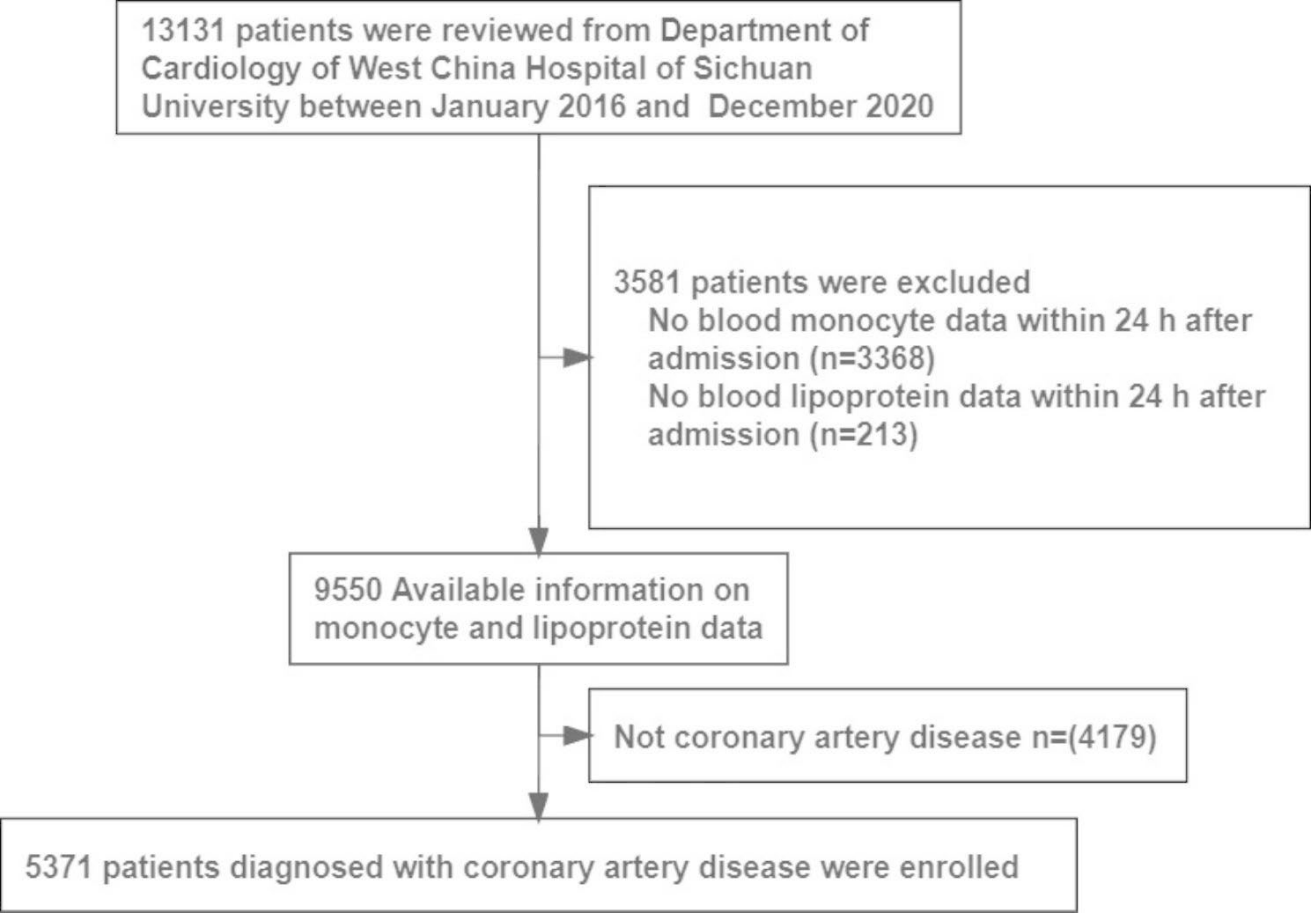
Flow chart

In total, 5371 patients were included (4035 men and 1336 women; mean age 66.13 ± 12.77 years) **(**Fig. 1**)**. The mean MHR value was 0.51 ± 0.37 (median/IQR: 0.42/0.28–0.62) and the low, medium, and high tertile distributions were 0.01 to 0.33 (T_1_), 0.33 to 0.54 (T_2_), and 0.54 to 4.98 (T_3_), respectively. During hospitalization, 114 (2.12%) patients died. Cardiovascular mortality accounted for 61.4% of all mortality: 52 patients had cardiogenic shock, 9 patients had cardiac rupture, 5 patients had heart failure, 3 patients had arrhythmia, 1 patient had aortic dissection. Non-cardiovascular mortality accounted for 38.6% of all mortality, with respiratory failure in 31 patients, MODs in 7 patients, infectious shock in 4 patients, and gastrointestinal bleeding in 2 patients. The rate of all-cause mortality increased in parallel from 1.95% in T_1_ and 1.96% in T_2_ to 6.65% in T_3_ (p < 0.001) with the increasing MHR levels.

Patients with higher MHR were younger and had higher levels of white blood cells, platelet counts, and blood glucose and lipids. MHR was inversely correlated with age (r= -0.123, p < 0.001), and positively correlated with white blood cells (r = 0.479, p < 0.001), platelet counts (r = 0.137, p < 0.001), glucose (r = 0.080, p < 0.001), and triglycerides (r = 0.076, p < 0.001). Patients in the higher MHR groups had a lower prevalence of hypertension, higher prevalence of ST-elevation myocardial infarction (STEMI), cardiogenic shock, diabetes and chronic kidney diseases (p < 0.05, Table 1).

**Table 1:** Baseline characteristics of included patients grouped by tertile of MHR

MHR tertile (%)	T_1_ (0.01–0.33) *	T_2_ (0.33–0.54)	T_3_(0.54–4.98)	P-value
N (%)	1793 (33.3)	1788 (33.3)	1790 (33.3)	-
Age, years, mean ± SD	68.00 ± 11.60	66.23 ± 12.64	64.14 ± 13.7	<0.001
Male, n (%)	1157 (64.53)	1413 (79.03)	1465 (81.84)	<0.001
SBP, mmHg, mean ± SD	129.75 ± 21.85	127.5 ± 22.49	122.45 ± 24.32	<0.001
DBP, mmHg, mean ± SD	75.40 ± 13.87	87.54 ± 303.89	75.18 ± 15.88	0.056
Breath, mean ± SD	19.91 ± 3.77	20.18 ± 4.87	20.3 ± 3.06	0.010
Hypertension, n (%)	864 (48.19)	835 (46.7)	768 (42.91)	0.005
Diabetes, n (%)	416 (23.2)	426 (23.83)	486 (27.15)	0.013
Hyperlipidemia, n (%)	1057 (58.95)	1088 (60.85)	1063 (59.39)	0.479
Atrial fibrillation, n (%)	166 (9.26)	137 (7.66)	189 (10.56)	0.011
Valvular heart disease, n (%)	75 (4.18)	64 (3.58)	70 (3.91)	0.646
Heart failure, n (%)	939 (52.37)	924 (51.68)	978 (54.64)	0.179
STEMI, n (%)	459 (25.60)	519 (29.03)	847 (47.32)	<0.001
NSTEMI, n (%)	208 (11.60)	299 (16.72)	371 (20.73)	<0.001
Cardiogenic shock, n (%)	24 (1.34)	24 (1.34)	86 (4.80)	<0.001
Stroke, n (%)	173 (9.65)	146 (8.17)	167 (9.33)	0.266
Tumor, n (%)	85 (4.74)	69 (3.86)	77 (4.3)	0.429
Chronic kidney diseases, n (%)	121 (6.75)	166 (9.28)	250 (28.2)	<0.001
COPD, n (%)	96 (5.35)	103 (5.76)	92 (5.14)	0.707
Current drinking, n (%)	539 (30.38)	646 (36.66)	707 (40.1)	<0.001
Current smoking, n (%)	646 (36.31)	822 (46.36)	988 (55.38)	<0.001
**Heart surgery**				
PCI, n (%)	990 (55.21)	1065 (59.56)	1170 (65.36)	<0.001
CABG, n (%)	369 (20.58)	354 (19.8)	160 (8.94)	<0.001
Valvular surgery, n (%)	31 (1.73)	18 (1.01)	11 (0.61)	0.006
Radiofrequency ablation, n (%)	45 (2.51)	32 (1.79)	19 (1.06)	0.005
**Complications**				
Infection, n (%)	183 (10.21)	220 (12.3)	438 (24.47)	<0.001
Pneumonia, n (%)	171 (9.54)	203 (11.35)	448 (25.03)	<0.001
Electrolyte disturbance, n (%)	62 (3.46)	76 (4.25)	155 (8.66)	<0.001
MOD, n (%)	1 (0.06)	2 (0.11)	15 (0.84)	<0.001
Disturbance of consciousness, n (%)	29 (1.62)	27 (1.51)	87 (4.86)	<0.001
Gastrointestinal bleeding, n (%)	27 (1.51)	33 (1.85)	71 (3.97)	<0.001
All-cause mortality, n (%)	35 (1.95)	35 (1.96)	119 (6.65)	<0.001
Cardiovascular mortality, n (%)	12 (0.67)	11 (0.62)	47 (2.63)	<0.001
Non-cardiovascular mortality, n (%)	9 (0.50)	9 (0.50)	26 (1.45)	0.001
**Laboratory tests**				
Monocytes, 10^9^/L, mean ± SD	0.30 ± 0.10	0.48 ± 0.13	0.78 ± 0.31	<0.001
HDL, mmol/L, mean ± SD	1.34 ± 0.35	1.12 ± 0.28	0.94 ± 0.28	<0.001
MHR, 10^9^/mmol, mean ± SD	0.23 ± 0.07	0.43 ± 0.06	0.87 ± 0.42	<0.001
White blood cells, 10^9^/L, mean ± SD	6.6 ± 2.7	7.77 ± 3.16	10.71 ± 4.76	<0.001
Hemoglobin, g/L, mean ± SD	129.53 ± 20.22	133.02 ± 21.14	131.92 ± 23.20	<0.001
Platelet counts, 10^9^/L, mean ± SD	163.77 ± 61.89	176.10 ± 76.78	191.96 ± 83.90	<0.001
Glucose, mmol/L, mean ± SD	7.68 ± 4.26	7.59 ± 3.73	8.27 ± 4.29	<0.001
Total cholesterol, mmol/L, mean ± SD	4.08 ± 1.16	3.95 ± 1.11	3.98 ± 1.23	<0.001
LDL, mmol/L, mean ± SD	2.29 ± 1.02	2.27 ± 0.97	2.36 ± 1.03	0.024
Triglyceride, mmol/L, mean ± SD	1.47 ± 1.1	1.69 ± 1.24	1.82 ± 1.44	<0.001

Abbreviation: MHR, monocytes to HDL ratio, T, tertile, SBP, systaltic blood pressure, DBP, diastolic blood pressure, COPD, chronic obstructive pulmonary disease, PCI, percutaneous coronary intervention, CABG, coronary artery bypass graft. MOD, multiple organ dysfunction syndrome, HDL, High-Density Lipoprotein, LDL, Low-Density Lipoprotein, NSTEMI, no ST-elevation myocardial infarction, STEMI, ST-elevation myocardial infarction, SD, standard deviation. * The range of MHR and the number of patients in each tertiles

### The relationship between MHR and all-cause mortality

When MHR was regarded as a continuous variable, univariable and multivariable analysis showed that increased MHR was associated with an enhanced rate of all-cause mortality. MHR per 1 unit increment was associated with a higher risk of all-cause mortality after adjusting for age and sex (model 1, HR 2.51, 95% CI [1.96, 3.21], p < 0.001, Table 2). Furthermore, after adjusting for age, sex, hypertension, diabetes, atrial fibrillation, renal failure, drinking, and smoking, MHR was independently associated with all-cause mortality (model 2, HR 1.81, 95% CI [1.35, 2.42], p < 0.001, Table 2). When MHR was regarded as a categorical variable, the adjusted HR (95% CI; P-value) for the highest tertile (T_3_) versus the lowest tertile (T_1_) was 2.30 (1.40–3.85; <0.001), with a dose-response relationship (P for trend < 0.001, Table 2).

**Table 2:** Cox regression analysis for mortality

	Univariate analysis HR (95%CI), P-value	Multivariate analysis (HR, 95%CI)	
		Model 1 h (95%CI), P-value	Model 2 h (95%CI), P-value
**All-cause mortality**			
MHR	2.16 (1.68,2.76), < 0.001	2.51 (1.96,3.21), < 0.001	1.81 (1.35, 2.42), < 0.001
MHR tertile			
T1	1	1	1
T2	0.89 (0.48, 1.68), 0.727	1.00 (0.53, 1.89), 0.990	0.77 (0.40, 1.50), 0.448
T3	2.87 (1.76, 4.69), < 0.001	3.49 (2.12, 5.75), < 0.001	2.30 (1.40, 3.85), 0.002
*P for trend*	< 0.001	< 0.001	< 0.001
**Cardiovascular mortality**			
MHR	2.19 (1.60,3.01), < 0.001	2.44 (1.78,3.34), < 0.001	1.69 (1.17, 2.45), 0.005
MHR tertile			
T1	1	1	1
T2	0.94 (0.41, 2.13), 0.882	0.99 (0.44, 2.26), 0.984	0.86 (0.38, 1.96), 0.719
T3	3.58 (2.44, 5.25), < 0.001	3.48 (1.81, 6.69), < 0.001	2.22 (1.14, 4.32), 0.019
*P for trend*	< 0.001	< 0.001	0.006
**Non-Cardiovascular mortality**			
MHR	2.10 (1.41,3.12), < 0.001	2.64 (1.78,3.91), < 0.001	2.04 (1.27, 3.28), 0.003
MHR tertile			
T1	1	1	1
T2	0.83 (0.31, 2.24), 0.718	1.03 (0.38, 2.79), 0.953	0.68 (0.22, 2.10), 0.500
T3	2.57 (1.20, 5.49), 0.015	3.53 (1.63, 7.67), 0.001	2.46 (1.08, 5.59), 0.032
*P for trend*	0.006	0.001	0.012

Model 1: adjusted for age and sex. Model 2: adjusted for age, sex, hypertension, diabetes, atrial fibrillation, chronic kidney diseases, drinking, smoking. Abbreviation: MHR, monocytes to HDL ratio, T, tertile, HR, hazard ratio, 95%CI, 95% confidence interval

**Fig. 2:**
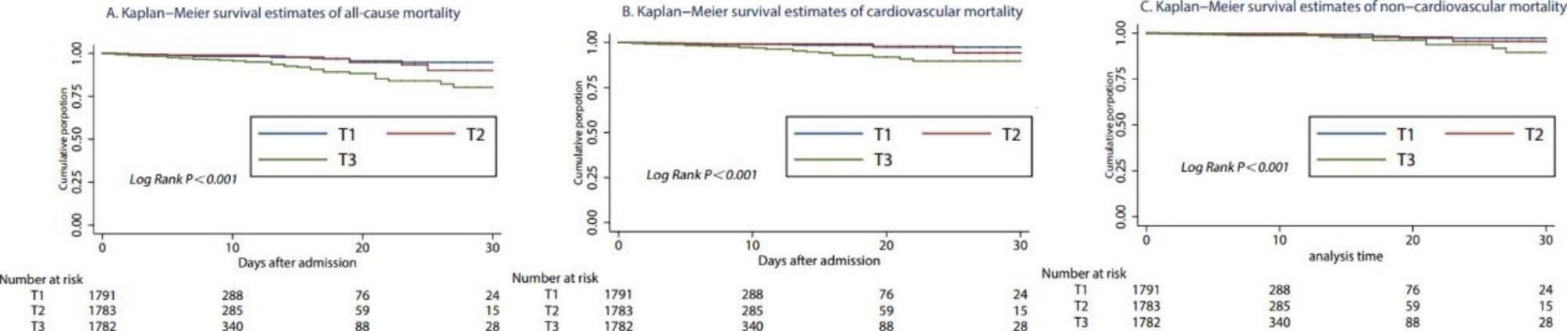
Kaplan-Meier survival analysis for all-cause mortality, cardiovascular mortality, and non-cardiovascular mortality of three groups

**Fig. 3:**
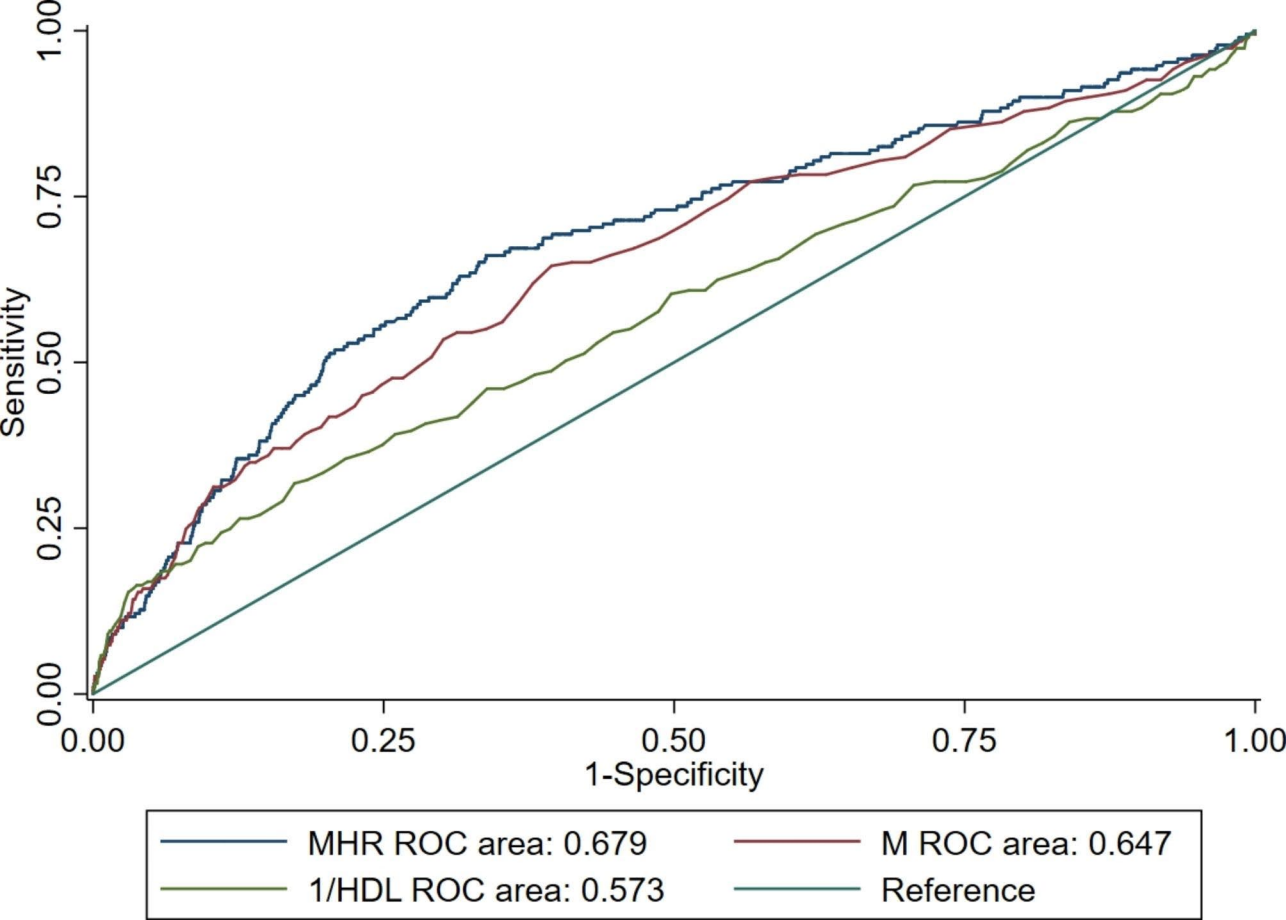
An area under the curve of receiver operating characteristic for monocytes, HDL, and MHR predicting in-hospital death

In addition, the MHR was positively associated with the risk of cardiovascular mortality and non-cardiovascular mortality regardless of whether the data of MHR were continuous or categorical (Table 2). Kaplan–Meier survival analysis suggested that patients in the T_3_ group had a significantly increased risk of all-cause mortality, cardiovascular mortality, and non-cardiovascular mortality compared with patients in the T_1_ and T_2_ groups (all log rank P < 0.001, Fig. 2). Based on the ROC curve, MHR values for predicting all-cause mortality yielded an AUC of 0.679 (95% CI, 0.637–0.722), which was higher than the AUC of monocytes (0.647, 0.604–0.691) and HDL (0.573, 0.526–0.620, Fig. 3). The optimal cutoff for predicting all-cause mortality was 0.53, with a sensitivity of 66% and specificity of 66%.

### Hypertension affects the relationship between the MHR and all-cause mortality

**Fig. 4:**
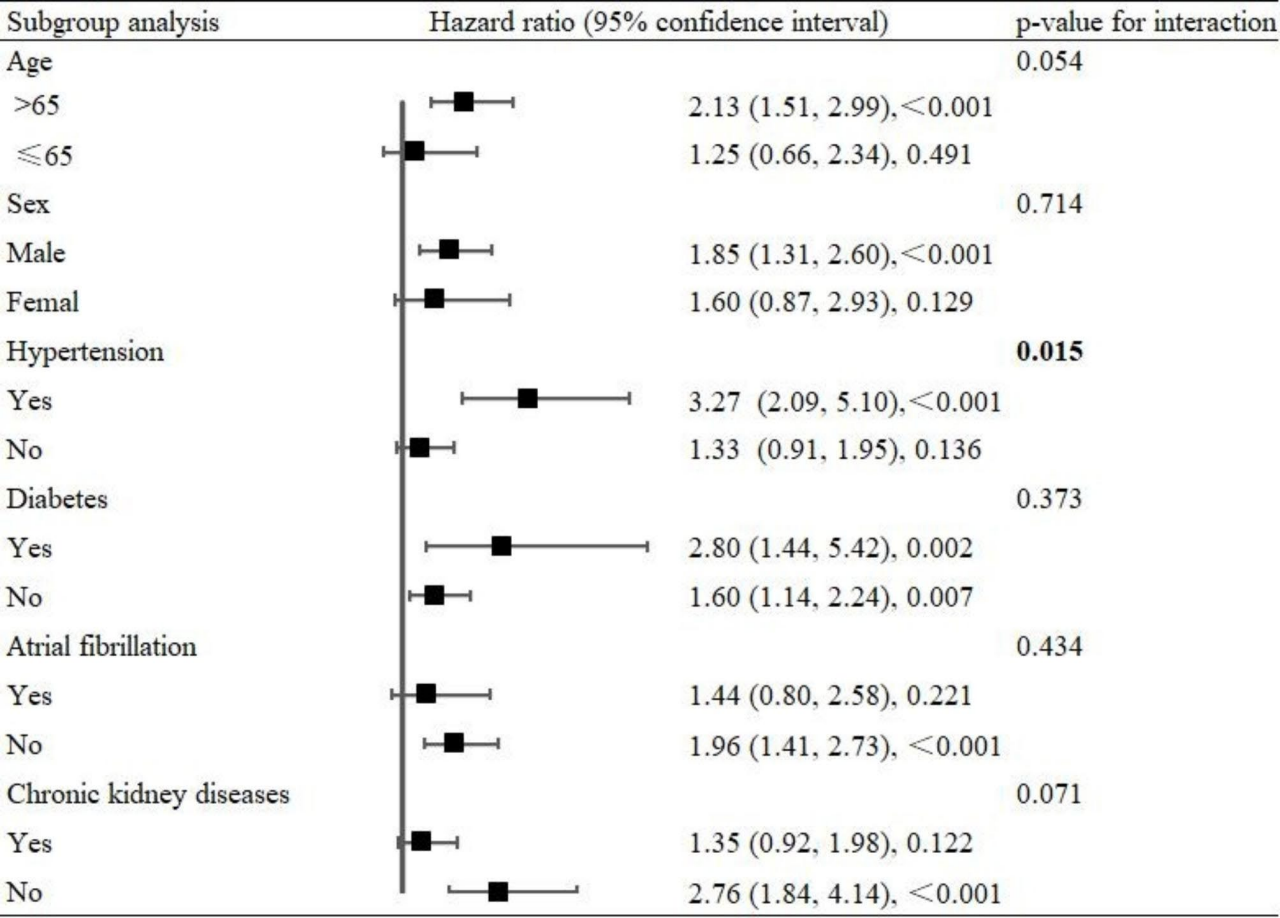
Stratified logistic regression analysis to identify variables that modify the correlation between (MHR) values and all-cause mortality. Adjusted factors included age, sex, hypertension, diabetes, atrial fibrillation, chronic kidney diseases, drinking, and smoking. The model was not adjusted for the stratification variable in each stratified analysis

Moreover, subgroup analysis also showed a significant interaction between MHR and hypertension (P for interaction = 0.003, Fig. 4). The association between the MHR and all-cause mortality was significant in patients with hypertension after adjusting for confounders (HR 3.27, 95% CI 2.09–5.10). However, this association was no longer significant in patients without hypertension (HR 1.33, 95% CI 0.91–1.95). For other stratified analyses, the association between the MHR and all-cause mortality was not altered by age, sex, diabetes, atrial fibrillation, or chronic kidney disease.

A higher MHR was also associated with a higher risk of in-hospital complications including infection, pneumonia, electrolyte disturbance, gastrointestinal bleeding, MODs, and disturbance of consciousness (Table 3). Additionally, we also found that the MHR was independently associated with all-cause mortality in patients receiving PCI, patients with acute coronary syndrome, and patients with different duration of CAD (onset to admission less than 1 month) (Tables 4 and 5).

**Table 3:** Cox regression analysis for complications

Complications	HR (95%CI), P-value
Infection	1.61 (1.43, 1.82), < 0.001
Pneumonia	1.66 (1.48, 1.87), < 0.001
Electrolyte disturbance	1.63 (1.33, 1.98), < 0.001
Gastrointestinal bleeding	1.63 (1.22, 2.19), 0.001
MOD	2.55 (1.50, 4.35), 0.001
Disturbance of consciousness	2.33 (1.86, 2.93), < 0.001

Adjusted for age, sex, hypertension, diabetes, atrial fibrillation, chronic kidney diseases, drinking, smoking. Abbreviation: MOD, multiple organ dysfunction syndrome. HR, hazard ratio, 95%CI, 95% confidence interval

**Table 4:** Cox regression analysis for all-cause mortality in patients receiving PCI or patients with ACS

	Univariate analysis HR (95%CI), P-value	Multivariate analysis (HR, 95%CI)	
		Model 1 h (95%CI), P-value	Model 2 h (95%CI), P-value
**PCI**			
MHR	1.85 (1.26,2.72), 0.002	2.23 (1.52,3.26), < 0.001	1.81 (1.18, 2.77), 0.007
MHR tertile			
T1	1	1	1
T2	1.08 (0.39, 2.99), 0.879	1.24 (0.45, 3.46), 0.673	1.15 (0.41, 3.22), 0.795
T3	2.94 (1.29, 6.68), 0.010	3.77 (1.64, 8.68), 0.002	2.93 (1.25, 6.88), 0.014
*P for trend*	0.003	< 0.001	0.004
**ACS**			
MHR	2.00 (1.47, 2.72), < 0.001	2.27 (1.70, 3.03), < 0.001	1.69 (1.24, 2.34), 0.001
MHR tertile			
T1	1	1	1
T2	0.82 (0.48, 1.80), 0.821	1.03 (0.53, 2.01), 0.936	0.77 (0.38, 1.55), 0.458
T3	1.89 (1.11, 3.20), 0.018	2.36 (1.38, 4.05), 0.002	1.84 (1.05, 3.22), 0.033
*P for trend*	0.006	< 0.001	0.008

Model 1: adjusted for age and sex. Model 2: adjusted for age, sex, hypertension, diabetes, atrial fibrillation, chronic kidney diseases, drinking, smoking. Abbreviation: MHR, monocytes to HDL ratio, PCI, percutaneous coronary intervention, ACS, acute coronary syndrome, T, tertile, HR, hazard ratio, 95%CI, 95% confidence interval

**Table 5:** Cox regression analysis for all-cause mortality in patients with different duration (less/more than 1 month)

	Univariate analysis HR (95%CI), P-value	Multivariate analysis (HR, 95%CI)	
		Model 1 h (95%CI), P-value	Model 2 h (95%CI), P-value
**Onset to admission less than 1 month**			
MHR	2.06 (1.44, 2.96), < 0.001	2.37 (1.70, 3.31), < 0.001	2.05 (1.40, 2.99), < 0.001
MHR tertile			
T1	1	1	1
T2	1.27 (0.53, 3.07), 0.595	1.46 (0.60, 3.58), 0.405	1.14 (0.43, 2.97), 0.791
T3	2.49 (1.20, 5.16), 0.014	3.34 (1.58, 7.06), 0.002	2.89 (1.31, 6.35), 0.008
*P for trend*	0.006	< 0.001	0.002
**Onset to admission more than 1 month**			
MHR	2.15 (1.50, 3.09), < 0.001	2.49 (1.71, 3.63), < 0.001	1.49 (0.94, 2.37), 0.087
MHR tertile			
T1	1	1	1
T2	0.59 (0.23, 1.51), 0.274	0.65 (0.25, 1.65), 0.364	0.51 (0.20, 1.32), 0.166
T3	2.99 (1.53, 5.85), 0.001	3.39 (1.72, 6.70), < 0.001	1.59 (0.78, 3.23), 0.203
*P for trend*	< 0.001	< 0.001	0.084

Model 1: adjusted for age and sex. Model 2: adjusted for age, sex, hypertension, diabetes, atrial fibrillation, chronic kidney diseases, drinking, smoking. Abbreviation: MHR, monocytes to HDL ratio, T, tertile, HR, hazard ratio, 95%CI, 95% confidence interval

## Discussion

In this study, the monocyte to high-density lipoprotein ratio was an independent predictor of all-cause mortality and in-hospital complications in patients with CAD, especially in patients with hypertension. Older age and poor heart function were the main predictors of in-hospital mortality [31], but whether other factors were also crucial for the short-term prognosis following CAD was unclear due to the complicated pathological mechanisms after disease onset. To provide additional evidence, we performed a cohort study with a relatively large sample size. Interestingly, the MHR had better prognostic values than monocytes and HDL as individual indicators. We found that the MHR was an independent predictor of mortality and complications in patients with CAD. Moreover, we also identified a positive interaction between the MHR and hypertension in CAD patients.

In this study, the cutoff values of MHR tertiles were 0.33 and 0.53, which were consistent with previous studies. They suggested that an MHR of 0.3 was the cutoff value for predicting coronary artery stenosis [32, 33]. Furthermore, previous studies suggested that the cutoff point of MHR was 0.53 for poor outcome, which was the same as our study [15]. In addition, the prognostic values in their study (AUC: 0.639; sensitivity: 60.5%; specificity: 65.6%) were similar to ours (AUC: 0.679; sensitivity: 66%; specificity: 66%). Of note, the mortality rate in our cohort (2.12%) was lower than the mortality rate (3 -14%) in other studies [15]. One possible reason may be that we included more male patients, who have lower mortality than female patients according to previous studies [34].

Furthermore, we found that patients with hypertension and elevated MHR may have a higher risk of all-cause mortality. Patients with hypertension have a higher risk of coronary stenosis, atherosclerosis, myocardial infarction, and cardiovascular mortality, indicating that hypertension might influence the progression and development of arteriosclerosis and coronary artery lesions [35]. High blood pressure as a physical force causes excess inflammation and endothelial impairment, which promote the development of arteriosclerotic plaque, as well as microvascular impairment [35, 36]. With chronic microvascular fragility, hypertension might account for earlier and greater coronary damage in CAD patients. Therefore, patients with a higher MHR and hypertension may have severe microvascular damage and arteriosclerosis, and thereby having a higher risk of all-cause mortality. In addition, several reports have shown that MHR could be used as a possible marker for plaque formation and severity, especially in diabetic patients [37–40]. However, there was no study which investigated the association between MHR and death in patients without diabetes. In our study, there was a significant association between MHR and mortality, in patients with diabetes (HR 2.81, 95%CI 1.44, 5.42) and in patients without diabetes (HR 1.60, 95%CI 1.14, 2.24). The interaction between MHR and diabetes was insignificant (P for interaction = 0.373). A possible explanation for this might be the lack of a more detailed data of plaque and coronary lesion severity in CAD patients with and without DM. This is an important issue for future research.

Monocytes are derived from myeloid progenitor cells in the bone marrow. After abnormal vascular endothelial function, activated monocytes interact with damaged endothelial cells, migrate to the subendothelial membrane and differentiate into macrophages. Macrophages phagocytose oxidized low-density lipoprotein particles to form foam cells [41, 42], and the foam cells with lipid streaks can secrete pro-inflammatory factors, thereby stimulating the inflammatory response around the damaged blood vessels, promoting the decomposition of the elastic membrane in the blood vessels, participating in chronic inflammation and the occurrence and development of atherosclerosis and plaque rupture [43]. Excess monocytes in peripheral blood impaired myocardial function [44]. Thus, patients with elevated monocytes may have poor outcomes.

Nevertheless, as a promoter to reverse cholesterol transport from cells back to the liver, HDL was reported to play a beneficial role after coronary heart diseases in anti-inflammation and decreasing oxidative stress by regulating cholesterol efflux and the function of monocytes [45]. HDL inhibits the production of monocytes by suppressing interleukin-23 and granulocyte-colony stimulating factor, thereby achieving an anti-inflammatory effect [46]. It can also regulate the activation, adhesion and migration of monocytes, preventing monocyte migration to the vascular subendothelial membrane [47]. HDL can neutralize the pro-inflammatory and pro-oxidative effects of monocytes, thereby preventing monocytes from adhering to the vessel wall, and protecting endothelial cells from inflammatory responses and oxidative stress damage [48]. Additionally, it has been reported that HDL and its major protein component, apolipoprotein A-1, have anti-inflammatory effects on monocytes by preventing CD11b activation [49]. The decrease in HDL may contribute to the instability of atherosclerotic plaque and disease deterioration [50].

It is still unknown why MHR is associated with short-term outcomes in patients with CAD, but there are several plausible explanations for the underlying biological mechanisms. First, an elevated MHR increases the risk of death, probably by increasing atherosclerosis progression, necrotic core rupture and thrombus formation [51]. Monocytes play an important role in the development of atherosclerosis [52]. HDL acts as an anti-atherosclerotic lipoprotein, preventing cholesterol transport to the arterial wall, especially in lipid-laden macrophages [53, 54]. Therefore, monocytes and HDL are independent predictors of atherosclerotic plaque regression [55, 56]. Besides, the higher rate of complications during hospitalization in patients with an increased MHR may be another reason. We found that the MHR was positively correlated with white blood cells. The elevated MHR may reflect the degree of inflammation. The high levels of inflammation and oxidative stress may lead to a higher risk of adverse events such as infection, MOD, gastrointestinal bleeding, and disturbance of consciousness in patients with CAD [10, 57, 58]. Thus, the MHR may be a helpful indicator reflecting the severity of inflammation, and the occurrence of complications to recognize patients with a high risk of mortality.

Previous studies have revealed positive associations between MHR and disease severity [7, 59], myocardial bridge [60], in-hospital major adverse cardiovascular events [61], and in-hospital and five-year mortality [16]. Our study was consistent with these studies, and we also found that the MHR was associated with mortality not only in patients with certain types of CAD. Specially, consistent with previous study [62], this research found that patients in the higher MHR groups had higher prevalence of STEMI (T1, 25.60%; T2, 29.03%; T3, 47.32%; p<0.001) (Table 1). To the best of our knowledge, no study has explored the relationship between the MHR and non-cardiovascular mortality as well as complications such as infection and MODs. We provide new information about the prognostic value of the MHR on non-cardiovascular mortality and complications. In addition, our results extended our knowledge regarding whether the MHR index has a superior predictive value for all-cause mortality than monocytes or HDL as a single index alone. From the perspective of clinical application, MHR may be useful for clinicians to categorize patients with a high risk of death and monitor disease severity as well as the inflammatory degree. Besides, future studies should determine whether novel therapies targeting MHR, such as anti-inflammatory drugs and statin medication decrease the risk of poor outcomes in patients with CAD.

Although the present cohort study had the largest sample size among previously published studies, some limitations should be considered. We only collected the baseline data of MHR, and data at multiple time points may provide more dynamic information. In addition, we only detected the count of total monocytes, and did not distinguish the subtypes of monocytes. Different subtypes of monocytes may play different roles in the prognosis of CAD. Future research should subdivide the different subtypes of monocytes to better explore the role of monocytes [63].

In conclusion, this study shows that a high level of MHR was an independent predictor of short-term prognosis in CAD patients, was closely related to the degree of atherosclerosis, and may serve as an important predictor of poor prognosis in CAD patients. MHR is a relatively simple and convenient biomarker. Therefore, monitoring MHR level may be of great significance to identify patients with high risk of short-term death, and improve the survival rate. This new marker could serve as an inexpensive and readily available risk stratification tool. Further studies should be conducted to assess its clinical utility in CAD patients.

## Conclusion

MHR was an independent predictor of all-cause mortality and in-hospital complications in patients with CAD, especially in patients with hypertension.

## Data Availability

The datasets used and/or analyzed during the current study are available from the corresponding author on reasonable request.
